# Safety Evaluation in Iterative Development of Wearable Patches for Aripiprazole Tablets With Sensor: Pooled Analysis of Clinical Trials

**DOI:** 10.2196/44768

**Published:** 2023-12-12

**Authors:** Michael Jan, Antonia Coppin-Renz, Robin West, Christophe Le Gallo, Jeffrey M Cochran, Emiel van Heumen, Michael Fahmy, J Corey Reuteman-Fowler

**Affiliations:** 1 Otsuka Pharmaceutical Development & Commercialization, Inc Princeton, NJ United States; 2 Otsuka Pharma GmbH Frankfurt am Main Germany; 3 Genmab US, Inc Plainsboro, NJ United States

**Keywords:** wearable sensor, adhesive patch, adverse events, skin irritation, product iteration, mobile phone, biocompatibility, Abilify MyCite, development, sensors, skin, monitoring, treatment, schizophrenia, bipolar disorder, depressive disorder, abrasions, blisters, dermatitis, pain, rash

## Abstract

**Background:**

Wearable sensors in digital health may pose a risk for skin irritation through the use of wearable patches. Little is known about how patient- and product-related factors impact the risk of skin irritation. Aripiprazole tablets with sensor (AS, Abilify MyCite; Otsuka America Pharmaceutical, Inc) is a digital medicine system indicated for the treatment of patients with schizophrenia, bipolar I disorder, and major depressive disorder. AS includes aripiprazole tablets with an embedded ingestible event marker, a wearable sensor attached to the skin through a wearable patch, a smartphone app, and a web-based portal. To continuously improve the final product, successive iterations of wearable patches were developed, including raisin patch version 4 (RP4), followed by disposable wearable sensor version 5 (DW5), and then reusable wearable sensor version 2 (RW2).

**Objective:**

This analysis pooled safety data from clinical studies in adult participants using the RP4, DW5, and RW2 wearable patches of AS and evaluated adverse events related to the use of wearable patches.

**Methods:**

Safety data from 12 studies in adults aged 18-65 years from May 2010 to August 2020 were analyzed. All studies evaluated safety, with studies less than 2 weeks also specifically examining human factors associated with the use of the components of AS. Healthy volunteers or patients with schizophrenia, bipolar I disorder, or major depressive disorder were enrolled; those who were exposed to at least 1 wearable patch were included in the safety analysis. Adverse events related to the use of a wearable patch were evaluated. Abrasions, blisters, dermatitis, discoloration, erythema, irritation, pain, pruritus, rash, and skin reactions were grouped as skin irritation events (SIEs). All statistical analyses were descriptive.

**Results:**

The analysis included 763 participants (mean [SD] age 42.6 [12.9] years; White: n=359, 47.1%; and male: n=420, 55%). Participants were healthy volunteers (n=269, 35.3%) or patients with schizophrenia (n=402, 52.7%), bipolar I disorder (n=57, 7.5%), or major depressive disorder (n=35, 4.6%). Overall, 13.6% (104/763) of the participants reported at least 1 SIE, all of which were localized to the wearable patch site. Incidence of ≥1 patch-related SIEs was seen in 18.1% (28/155), 14.2% (55/387), and 9.2% (28/306) of participants who used RP4, DW5, and RW2, respectively. Incidence of SIE-related treatment discontinuation was low, which is reported by 1.9% (3/155), 3.1% (12/387), and 1.3% (4/306) of participants who used RP4, DW5, and RW2, respectively.

**Conclusions:**

The incidence rates of SIEs reported as the wearable patch versions evolved from RP4 through RW2 suggest that information derived from reported adverse events may have informed product design and development, which could have improved both tolerability and wearability of successive products.

**Trial Registration:**

Clinicaltrials.gov NCT02091882, https://clinicaltrials.gov/study/NCT02091882; Clinicaltrials.gov NCT02404532, https://clinicaltrials.gov/study/NCT02404532; Clinicaltrials.gov NCT02722967, https://clinicaltrials.gov/study/NCT02722967; Clinicaltrials.gov NCT02219009, https://clinicaltrials.gov/study/NCT02219009; Clinicaltrials.gov NCT03568500, https://clinicaltrials.gov/study/NCT03568500; Clinicaltrials.gov NCT03892889, https://clinicaltrials.gov/study/NCT03892889

## Introduction

Wearable sensors and other digital health tools have the potential to improve health care in multiple disease areas, including mental illness [1], oncology [2], cardiovascular disorders [3], and diabetes [4]. Unlike biomedical implants, wearable sensors are minimally invasive and can be easily worn making them convenient for patients’ daily use [5]. With objective and continuous health monitoring, wearable sensors also enable the comprehensive collection of standardized patient information [1,6,7], facilitate informed health care decision-making [8], and assist chronic disease management [7]. However, prolonged use of wearable sensors through the use of wearable patches raises safety concerns such as allergic reactions and skin irritation [5,9,10]. For example, wearable patches may contain metals, adhesives, and rubber components that pose a risk for skin irritation [11]. Thus, biocompatibility considerations are a priority in the development of wearable patches [12].

Biocompatibility is defined as the ability of a medical device material to perform with an appropriate host response in a specific application [13]. It allows a medical device or biomaterial to be accepted by the surrounding tissue and human body and prevents undesirable responses such as allergic reactions and chronic inflammation [14]. The justification of biocompatibility is required by the US Food and Drug Administration (FDA) and the European Union for any medical device with direct or indirect tissue contact [13,15,16]. Wearable sensors are devices worn on the human body or clothing [17]; therefore, wearable sensors intended for medical use are regulated as medical devices in the United States and the European Union [16,18]. Despite the emerging potential of wearable sensors in health care, most wearable sensors are not cleared by the FDA or approved by the European Union. In a review of 362 wearable sensors, only 21 (5.7%) attained regulatory approval in the US or European markets, indicating a lack of regulatory-grade data for clinical decision-making [19].

Aripiprazole tablets with sensor (AS, Abilify MyCite) is an FDA-approved digital medicine system indicated for patients with schizophrenia, bipolar I disorder, and major depressive disorder [20]. One of the major challenges for patients with psychotic disorders is poor adherence to therapy [21], which has been associated with higher rates of violence, hospital admission, substance use disorders, and increased risk of relapse and mortality [22-24]. AS includes aripiprazole tablets with an embedded ingestible event marker, a wearable sensor attached to the skin through a wearable patch, a smartphone app, and a web-based portal [20]. The ingestible event marker embedded within the medication tablet is activated by stomach fluid after ingestion and communicates with the nonmedicated wearable patch [25-27]. The wearable sensor receives the signal from the ingestible sensor and sends the information to the smartphone app. The data can be accessed by patients via the smartphone app or by health care providers and caregivers through a web portal. In a recent phase 3b multicenter, prospective, open-label trial, AS was associated with a significant reduction in inpatient psychiatric hospitalization rates for adults with mild to moderate schizophrenia compared with standard-of-care antipsychotics [28]. Clinical data also suggest that AS was well tolerated, with skin irritation reported as the most common treatment-emergent adverse events (TEAEs) in some trials [25,28-33].

**Figure 1:**
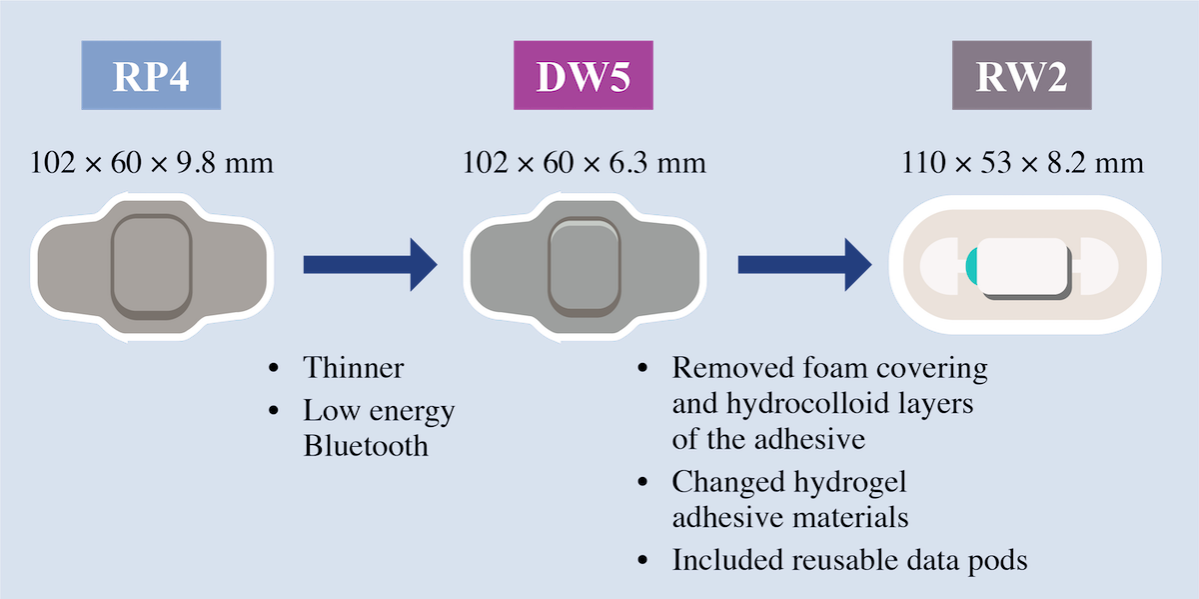
Iteratively developed wearable patches based on participant feedback. DW5: disposable wearable sensor version 5; RP4: raisin patch version 4; RW2: reusable wearable sensor version 2.

Irritation is an important biocompatibility end point in the evaluation of medical devices [13]. However, there are gaps in our understanding of how patient-related factors (eg, skin sensitivity and weight) and patch-related factors (ie, composition of wearable patch material, dimensions, duration of use, and frequency of wearable patch changes) impact the risk of skin irritation. Product safety was discussed routinely alongside product quality, product engineering, and technical operations as part of failure mode and effects analysis (FMEA) for the development of the wearable patch component of AS. In addition to hypothetical process failures, the FMEA of the wearable patch component of AS was informed by events observed in the postmarketing setting and in clinical and human factors trials. To continuously improve the final product, successive iterations of the wearable patches have been developed for AS, including raisin patch version 4 (RP4), followed by disposable wearable sensor version 5 (DW5), and then reusable wearable sensor version 2 (RW2), which is the most recent version. In addition to technical improvements, product design changed as wearable patch versions evolved to address issues observed with previous wearable patches to improve comfort, wearability, and usability (Figure 1). For example, in the development of the DW5, the thickness of the wearable patch was reduced from that of the RP4 to improve wearability under clothing based on user feedback. Similarly, in the development of the RW2, the foam covering and hydrocolloid layers were removed to improve comfort and breathability based on human factor studies. Additionally, the hydrogel adhesive material was changed from that used in the predecessor DW5 to reduce the potential for skin irritation. To improve usability, the 2-component design of the RW2 included a reusable data pod that reduced the user’s burden of needing to pair each new wearable patch with the mobile phone app [34]. Leveraging participant experiences from a large pool of clinical trial data, we evaluated safety data across 12 company-sponsored studies on the wearable patches of AS. Here, we report skin irritation events (SIEs) after the use of these 3 wearable patch versions in study participants.

## Methods

### Data Source and Participants

Safety data were pooled from 12 clinical trials in adults using the wearable patch component of AS from May 2010 to August 2020 (trial identifiers [trial registration numbers]: 316-13-204 [Otsuka], 316-13-205 [Otsuka], 316-13-206A [NCT02091882], 316-13-206B [NCT02091882], 316-13-215 [NCT02722967], 316-14-220 [NCT02219009], 031-201-00186 [NCT03568500], 031-201-00266 [Otsuka], 031-201-00301 [NCT03892889], 031-201-00383 [Otsuka], 031-201-00420 [Otsuka], and 031-201-00469 [Otsuka]). Participants were enrolled from hospitals and real-world settings and were screened per inclusion or exclusion criteria for each study. In some studies, participants were excluded if unable to use wearable patches due to known allergies to adhesives or dermatological issues such as active skin infection, active dermatitis, or chronic inflammatory skin conditions (eg, psoriasis). The duration of the clinical trials ranged from 1 day to 6 months. Six short-term studies were less than 2 weeks, and 6 long-term studies were at least 4 weeks; there were no studies with a length between 2 and 4 weeks (Multimedia Appendix 1). In short-term studies, participants wore 1 or multiple wearable patches simultaneously on distinct parts of the abdomen for the whole study duration without replacement of wearable patches. In long-term studies, participants wore 1 wearable patch at a time and were instructed to replace the wearable patch every 7 days. All studies evaluated the safety of the wearable patch component of AS. Additionally, long-term studies evaluated the efficacy and usability of AS, and short-term studies examined human factors associated with the use of components of AS. Those who were exposed to at least 1 wearable patch were included in the pooled safety analysis [25,28-33]. Eligible participants were aged 18-65 years and were either healthy volunteers or had received a diagnosis of schizophrenia, bipolar I disorder, or major depressive disorder.

### Ethical Considerations

Studies were conducted in accordance with local laws, the International Conference on Harmonization Good Clinical Practice guidelines, and the Declaration of Helsinki and were approved by individual institutional review boards or independent ethics committees at each participating center. All participants gave signed and dated informed consent prior to inclusion in the respective trials; the informed consent allowed for secondary analysis without additional consent. Study data were anonymized or deidentified. All 12 studies reimbursed participants for their time and travel, and no additional stipend was provided. All 12 studies included in this analysis allow for secondary analysis without additional IRB approval.

### Skin Irritation Events

TEAEs related to the use of a wearable patch were classified using Medical Dictionary for Regulatory Activities Terminology v24.0 preferred terms. Abrasions, blisters, dermatitis, discoloration, erythema, irritation, pain, pruritus, rash, and skin reactions were grouped as SIEs.

### Statistical Analysis

This pooled analysis aimed to characterize the incidence of SIEs with iterative improvements in wearable patch design. All statistical analyses were descriptive. In the intention-to-treat analysis, SIE incidence for each type of wearable patch was assessed by unique study participants in all 12 studies (6 short-term and 6 long-term studies) and by duration of wearable patch use in 4 long-term studies. When participants used more than 1 type of wearable patch, SIEs were counted separately for each participant by patch combination. Treatment discontinuation due to SIEs was tabulated by patch type across all studies. SIE incidence by the participant was calculated as the number of participants with SIEs divided by the total number of participants × 100%. SIE incidence by duration of wearable patch use was calculated as total SIEs divided by the total number of days of wearable patch use for all participants × 365.25.

## Results

### Participant Characteristics

Overall, 155, 387, and 306 participants were exposed to the iteratively developed wearable patches RP4, DW5, and RW2, respectively. From short-term studies 031-201-00383 (39 participants; Otsuka) and 031-201-00420 (46 participants; Otsuka), a total of 85 participants were exposed to multiple wearable patches simultaneously on distinct parts of the abdomen. Thus, data from a total of 763 unique participants, who were exposed to at least 1 wearable patch, were analyzed (Table 1). The mean (SD) age of participants wearing any wearable patch was 42.6 (12.9) years. Most participants had received a diagnosis of schizophrenia (n=402, 52.7%) or were healthy volunteers (n=269, 35.3%), were White (n=359, 47.1%) or African American (n=333, 43.6%), and were male (n=420, 55%).

**Table 1:** Participant demographics.

Parameter		RP4^a^ (n=155)	DW5^b^ (n=387)	RW2^c^ (n=306)	Any wearable patch^d^ (n=763)
Studies, n		4	7	5	12
Age (years), mean (SD)		42.2 (13.0)	42.2 (13.0)	42.5 (13.0)	42.6 (12.9)
**Sex, n (%)**					
	Male	70 (45.2)	227 (58.7)	163 (53.3)	420 (55.0)
	Female	85 (54.8)	160 (41.3)	143 (46.7)	343 (45.0)
**Race or ethnicity, n (%)**					
	White	97 (62.6)	166 (42.9)	137 (44.8)	359 (47.1)
	Black or African American	40 (25.8)	183 (47.3)	143 (46.7)	333 (43.6)
	American Indian or Alaskan Native	1 (0.6)	3 (0.8)	3 (1.0)	7 (0.9)
	Asian	7 (4.5)	24 (6.2)	16 (5.2)	40 (5.2)
	Native Hawaiian or Pacific Islander	1 (0.6)	0 (0.0)	0 (0.0)	1 (0.1)
	Other	9 (5.8)	11 (2.8)	7 (2.3)	23 (3.0)
**Clinical characteristics, n (%)**					
	Schizophrenia	37 (23.9)	239 (61.8)	126 (41.2)	402 (52.7)
	Major depressive disorder	23 (14.8)	12 (3.1)	0 (0.0)	35 (4.6)
	Bipolar I disorder	35 (22.6)	22 (5.7)	0 (0.0)	57 (7.5)
	Healthy volunteers^e^	60 (38.7)	114 (29.5)	180 (58.8)	269 (35.3)

^a^RP4: raisin patch version 4.

^b^DW5: disposable wearable sensor version 5.

^c^RW2: reusable wearable sensor version 2.

^d^Eighty-five participants were exposed to multiple wearable patches; thus, the sum of the RP4, DW5, and RW2 counts may not be equal to any wearable patch counts.

^e^No serious mental illness diagnosis.

### Incidence of SIEs by Wearable Patch Type

Across all the clinical trials, 13.6% (104/763) of participants experienced at least 1 SIE (Table 2, Multimedia Appendix 2). All SIEs were localized to the wearable patch site (ie, application or medical device site). Stratified by patch type, at least 1 SIE was experienced by 18.1% (28/155), 14.2% (55/387), and 9.2% (28/306) of participants who used RP4, DW5, and RW2 wearable patches, respectively (Table 2).

**Table 2:** Participants who reported SIEs^a^ by wearable patch version.

			RP4^b^ (n=155)		DW5^c^ (n=387)		RW2^d^ (n=306)	Any wearable patch^e^ (n=763)
Any SIEs, n (%)			28 (18.1)		55 (14.2)		28 (9.2)	104 (13.6)
**Application site,^f^ n (%)**								
	Erythema	4 (2.6)		0 (0)		0 (0)		4 (0.5)
	Pain	1 (0.6)		0 (0)		0 (0)		1 (0.1)
	Pruritus	6 (3.9)		0 (0)		0 (0)		6 (0.8)
	Rash	4 (2.6)		0 (0)		0 (0)		4 (0.5)
**Medical device site,^f^ n (%)**								
	Erythema	0 (0)		1 (0.3)		6 (2)		6 (0.8)
	Irritation	0 (0)		16 (4.1)		4 (1.3)		20 (2.6)
	Pruritus	0 (0)		8 (2.1)		15 (4.9)		17 (2.2)
	Rash^g^	0 (0)		6 (1.6)		4 (1.3)		10 (1.3)
	Reaction	0 (0)		1 (0.3)		2 (0.7)		2 (0.3)
**Rash,^g^ n (%)**								
	Erythematous	2 (1.3)		0 (0)		0 (0)		2 (0.3)
	Papular	1 (0.6)		1 (0.3)		0 (0)		2 (0.3)
	Pruritic	0 (0)		1 (0.3)		0 (0)		1 (0.1)
	Not specified	3 (1.9)		11 (2.8)		0 (0)		14 (1.8)
**Other SIEs, n (%)**								
	Blister	1 (0.6)		0 (0)		0 (0)		1 (0.1)
	Contact dermatitis	3 (1.9)		1 (0.3)		0 (0)		4 (0.5)
	Erythema	2 (1.3)		4 (1)		0 (0)		6 (0.8)
	Pruritus^h^	8 (5.2)		6 (1.6)		0 (0)		14 (1.8)
	Skin abrasion	1 (0.6)		0 (0.0)		0 (0)		1 (0.1)
	Skin discoloration	0 (0)		1 (0.3)		0 (0)		1 (0.1)
	Skin hyperpigmentation	0 (0)		1 (0.3)		0 (0)		1 (0.1)
	Skin irritation	0 (0)		2 (0.5)		0 (0)		2 (0.3)

^a^SIE: skin irritation event.

^b^RP4: raisin patch version 4.

^c^DW5: disposable wearable sensor version 5.

^d^RW2: reusable wearable sensor version 2.

^e^Eighty-five participants were exposed to multiple patches and experienced events with different patches; thus, the sum of the RP4, DW5, and RW2 counts may not be equal to any wearable patch counts.

^f^SIEs were coded as “Application site” for RP4, and as “Medical device site” for DW5 and RW2.

^g^Adverse events reported from earlier clinical trials with RP4 and DW5 were coded to specify types of rash and evolved to specify “Medical device site” in later trials with DW5 and RW2.

^h^The Medical Dictionary for Regulatory Activities Terminology preferred term coding evolved from “Pruritus” to “Application site pruritus” and then to “Medical device site pruritus.”

### SIE-Related Treatment Discontinuation

Across all 12 studies, treatment withdrawal due to SIEs was low. SIE-related treatment discontinuation occurred in 1.9% (3/155), 3.1% (12/387), and 1.3% (4/306) of participants who used RP4, DW5, and RW2 wearable patches, respectively.

### Incidence of SIEs by Study Length

Because the 12 trials were of different lengths, SIEs were stratified by the 6 long-term and 6 short-term studies (Multimedia Appendix 1). Incidence of SIEs was higher in long-term studies than in short-term studies. In long-term studies, 15.8% (83/524) of participants who used any wearable patch experienced at least 1 SIE (Table 3), while this number was 8.8% (21/239) in short-term studies.

In long-term studies, 125, 273, and 126 participants were exposed to RP4, DW5, and RW2, respectively. The percentage of participants who reported no SIEs increased with iteratively developed wearable patch versions. No SIEs were observed from long-term studies in 77.6% (97/125), 82.4% (225/273), and 94.4% (119/126) of participants who used RP4, DW5, and RW2 wearable patches, respectively. Among participants who reported SIEs, the incidence decreased as new versions of the wearable patch were developed. One SIE occurred in 18.4% (23/125), 15% (41/273), and 4.8% (6/126) of participants who used RP4, DW5, and RW2 wearable patches, respectively; 2 or more SIEs occurred in 4% (5/125), 2.6% (7/273), and 0.8% (1/126) of participants who used RP4, DW5, and RW2 wearable patches, respectively (Table 3).

**Table 3:** Participants experiencing SIEs^a^ in long-term studies.^b^

Participants with SIEs, n (%)			RP4^c^ (n=125)		DW5^d^ (n=273)		RW2^e^ (n=126)		Any wearable patch (n=524)
**At least 1 SIE**			28 (22.4)		48 (17.6)		7 (5.6)		83 (15.8)
	1 SIE	23 (18.4)		41 (15)		6 (4.8)		70 (13.4)	
	More than 1 SIE	5 (4)		7 (2.6)		1 (0.8)		13 (2.5)	

^a^SIE: skin irritation event.

^b^Long-term studies were those of ≥4 weeks' duration.

^c^RP4: raisin patch version 4.

^d^DW5: disposable wearable sensor version 5.

^e^RW2: reusable wearable sensor version 2.

### Incidence of SIEs by Duration of Wearable Patch Use

SIEs were also assessed based on the duration of wearable patch use in studies that have available data (Multimedia Appendices 3 and 4). The incidence by duration of wearable patch use in long-term studies with available data was 3.18, 1.59, 1.32, and 1.72 SIEs per person-year for RP4, DW5, RW2, and any wearable patch, respectively (Multimedia Appendix 5).

## Discussion

### Principal Findings

To our knowledge, this is the first pooled safety analysis to characterize adverse event reporting with iterative improvements in the design of wearable patches. Safety data were pooled from 12 clinical trials to analyze SIEs from the wearable patch component of AS. Although safety data have been published from some trials, this pooled analysis included additional data that have not been published before, from Otsuka clinical trials (316-13-204, 316-13-205, 031-201-00266, 031-201-00383, 031-201-00420, and 031-201-00469). Previously published studies were not comparative and did not comment on safety across studies and wearable patch design changes [25,28,29,31-33].

In parallel to long-term clinical trials that focused on determining the efficacy and usability of AS, short-term clinical trials examined human factors associated with the use of components of AS. As part of the FMEA of the wearable patch component of AS, events related to product safety as observed in real-world settings and in clinical and human factors studies were discussed regularly, and opportunities for design enhancement and potential risk mitigation were identified. To improve patient comfort, enhance ease of use, and reduce potential risk for skin irritation, RP4, DW5, and RW2 wearable patches were developed iteratively and were tested from May 2010 to August 2020. Observed SIE incidence rates with the optimization of the wearable patch design from RP4 through RW2 might suggest that information from reported adverse events may be incorporated into product evolution to develop better-tolerated and more-wearable products. This patient-centered approach addresses patients’ needs for better wearability and may yield benefits by encouraging longer use of the product to better align with the disease needs. Overall, this analysis highlights the importance of using safety findings from clinical studies to drive further product development and evaluate product performance in a setting of patient use.

Prolonged contact with a sensor that is attached to the skin through a wearable patch can raise concerns about skin irritation [5,9,10,20]. Prior clinical studies of AS reported that skin irritation was the main category of adverse events for AS [28,29,31,32]. In a recent phase 3b clinical trial of AS, the most common TEAEs were related to wearable patch use (7.6%) [28]. Earlier studies suggested incidences of SIEs ranging from 0% to 34.7% [25,31-33]. Consistent with these data, in this pooled analysis, SIEs across the 3 wearable patch versions were localized to the wearable patch site (ie, application or medication device site). For the most recently developed RW2 wearable patch, the incidence of SIEs was 9.2% (28/306), which was numerically lower than the incidence for the earlier patch versions, the DW5 (55/387, 14.2%) and RP4 (28/155, 18.1%). This observation may be due in part to material changes in the development of the RW2 wearable patch, where the hydrogel adhesive was changed and a foam covering and hydrocolloid layer were removed when compared with the predecessor DW5 wearable patch. Pruritus was reported in varying incidences with the use of wearable patches, may follow from the overall observed trend of decreased SIE incidence, or reflects a tradeoff between skin irritation and comfort. This uncertainty may be attributable to a combination of the following: (1) the Medical Dictionary for Regulatory Activities Terminology preferred term coding evolved from “Pruritus” to “Application site pruritus” to “Medical device site pruritus” and (2) investigators may have reported “Pruritus” as “Irritation” in some early studies.

The available data suggest that the potential interaction effects of sequential wearable patch replacement in long-term studies may be minimal. The incidence of SIEs by the duration of wearable patch use was low, with 1.72 SIEs per person-year for participants who were exposed to at least 1 wearable patch in long-term studies. Given that only 2.5% (13/524) of participants in long-term studies experienced more than 1 SIE, the percentage of participants with repeat SIEs may also be low. In some short-term studies, participants were exposed to multiple wearable patches simultaneously for a continuous 10 days. No clear trends were observed in SIE incidence rates between short-term studies with the use of a single wearable patch and short-term studies with the use of multiple wearable patches (Multimedia Appendix 4), suggesting that simultaneous patch use may not have an impact on SIE incidence during the short-term observation.

A higher percentage of participants reported SIEs in long-term studies than in short-term studies, suggesting a potentially increased risk of SIEs due to long exposure to the wearable patch. Therefore, biocompatibility considerations are a priority in the future development of the wearable patch for long-term use. The major biocompatibility issues involve identifying and understanding patient- and patch-related factors that impact the risk of skin irritation. To continuously improve the final product, we developed successive iterations of wearable patches for AS. We were able to investigate risk factors for skin irritation in the wearable patch of AS and develop more wearable products through the evolution of the wearable patch design. These studies may facilitate further optimization of the wearable patch of AS as new biomaterials with higher biocompatibility become available in the future.

### Limitations

This safety analysis pooled data from 12 clinical trials, 6 of which were short-term studies. Data from short-term studies may provide limited insight into SIEs in clinical settings because patients with serious mental illness usually require long-term treatment, and SIEs may not be observed with short-term exposure to a wearable patch. Therefore, interpretation of SIE incidence from short-term studies should be interpreted accordingly. Short-term studies were analyzed with respect to unique participant exposure instead of the total duration of use. The incidence of SIEs in terms of the number of wearable patches used per participant could not be analyzed; these data were not available for RW2 from long-term studies and only some RP4 long-term studies had these data available. However, since participants were instructed to use wearable patches continuously for 1 week and were notified weekly for wearable patch replacements, the numbers of wearable patches used may be approximated from the lengths of the long-term studies. Additionally, participant characteristics were not assessed to identify whether SIEs were more likely in any subgroups of participants to avoid the risk of overstating any associations between participant demographics and the observed incidence rates of SIEs. This pooled analysis also did not discuss discontinuations due to reasons other than SIEs. The analysis of SIEs with respect to duration in long-term studies may be affected by such discontinuations. However, only a small number of discontinuations were identifiably related to the use of the wearable patch component of AS: “Non-adherence of patch to the skin” (2 participants in study 316-13-215), “Non-compliance with patch wearing” (4 participants in study 316-13-215), and “Technical problems” (3 participants in study 031-201-00186). Another limitation is that some studies excluded participants who could not use the wearable patch due to known allergies or dermatological issues, such as active skin infection, active dermatitis, or chronic inflammatory skin conditions (eg, psoriasis). These exclusion criteria may limit the generalizability of this study. Finally, as these were post hoc analyses and not powered for detection of statistically significant differences, descriptive statistics were used throughout and any observed differences were numeric.

### Conclusions

Prolonged contact with wearable patches can cause skin irritation; therefore, safety is important when designing products for patients. The iteratively developed wearable patch of AS highlights how TEAEs from clinical trials and feedback from clinical trial participants regarding comfort and ease of wear can be incorporated to drive further product development. It also highlights how patients’ needs for better wearability can be addressed in a patient-centered approach, which hopefully may encourage longer use of the product to better align with the disease needs.

## Appendix

Multimedia Appendix 1 Clinical trial duration.

Multimedia Appendix 2 Participants experiencing skin irritation events in each clinical trial.

Multimedia Appendix 3 Incidence of skin irritation events by duration of wearable patch use in long-term studies with available data.

Multimedia Appendix 4 Incidence of skin irritation events by duration of wearable patch use in short-term studies with available data.

Multimedia Appendix 5 Incidence of skin irritation events based on duration of wearable patch use in long-term studies with available data.

## Abbreviations

AS: Aripiprazole tablets with sensor
DW5: disposable wearable sensor version 5
FDA: Food and Drug Administration
FMEA: failure mode and effects analysis
IPD: individual participant data
RW2: reusable wearable sensor version 2
RP4: raisin patch version 4
SIE: skin irritation event
TEAE: treatment-emergent adverse event

## Data Availability

To submit inquiries related to Otsuka clinical research, or to request access to anonymized individual participant data (IPD) associated with any Otsuka clinical trial, please visit the Clinical Trial Data Transparency website [35]. For all approved IPD-access requests, Otsuka will share anonymized IPD on a remotely accessible data-sharing platform.

## References

[1] Hirschtritt ME, Insel TR (2018). Digital technologies in psychiatry: present and future. Focus (Am Psychiatr Publ).

[2] Onyeaka HK, Zambrano J, Longley RM, Celano CM, Naslund JA, Amonoo HL (2021). Use of digital health tools for health promotion in cancer survivors. Psychooncology.

[3] Kaushik A, Patel S, Dubey K (2020). Digital cardiovascular care in COVID-19 pandemic: a potential alternative?. J Card Surg.

[4] Fagherazzi G, Ravaud P (2019). Digital diabetes: perspectives for diabetes prevention, management and research. Diabetes Metab.

[5] Khatsenko K, Khin Y, Maibach H (2020). Allergic contact dermatitis to components of wearable adhesive health devices. Dermatitis.

[6] Sano A, Taylor S, McHill AW, Phillips AJ, Barger LK, Klerman E, Picard R (2018). Identifying objective physiological markers and modifiable behaviors for self-reported stress and mental health status using wearable sensors and mobile phones: observational study. J Med Internet Res.

[7] Kim J, Campbell AS, de Ávila Berta Esteban-Fernández, Wang J (2019). Wearable biosensors for healthcare monitoring. Nat Biotechnol.

[8] Hilty DM, Armstrong CM, Edwards-Stewart A, Gentry MT, Luxton DD, Krupinski EA (2021). Sensor, wearable, and remote patient monitoring competencies for clinical care and training: scoping review. J Technol Behav Sci.

[9] McAdams E, Krupaviciute A, Gehin C, Grenier E, Massot B, Dittmar A, Rubel P, Fayn J (2011). Wearable sensor systems: the challenges. Annu Int Conf IEEE Eng Med Biol Soc.

[10] Hwang I, Kim HN, Seong M, Lee SH, Kang M, Yi H, Bae WG, Kwak MK, Jeong HE (2018). Multifunctional smart skin adhesive patches for advanced health care. Adv Healthc Mater.

[11] Winston FK, Yan AC (2017). Wearable health device dermatitis: a case of acrylate-related contact allergy. Cutis.

[12] Wang L, Lou Z, Jiang K, Shen G (2019). Bio-multifunctional smart wearable sensors for medical devices. Adv Intell Syst.

[13] (2020). Use of International Standard ISO 10993-1, "biological evaluation of medical devices—part 1: evaluation and testing within a risk management process". U.S. Food and Drug Administration.

[14] Cvrček L, Horáková M, Thomas S, Mozetič M, Cvelbar U, Špatenka P, Praveen KM (2019). Plasma modified polymeric materials for implant applications (Chapter 14). Non-Thermal Plasma Technology for Polymeric Materials.

[15] Special considerations for 510(k)s. U.S. Food and Drug Administration.

[16] Regulation (EU) 2017/745 of the European Parliament and of the council. Official Journal of the European Union.

[17] Iqbal SMA, Mahgoub I, Du E, Leavitt MA, Asghar W (2021). Advances in healthcare wearable devices. NPJ Flex Electron.

[18] How to determine if your product is a medical device. U.S. Food and Drug Administration.

[19] Muzny M, Henriksen A, Giordanengo A, Muzik J, Grøttland A, Blixgård H, Hartvigsen G, Årsand E (2020). Wearable sensors with possibilities for data exchange: analyzing status and needs of different actors in mobile health monitoring systems. Int J Med Inform.

[20] (2020). Abilify MyCite®: prescribing information. Otsuka.

[21] Haddad PM, Brain C, Scott J (2014). Nonadherence with antipsychotic medication in schizophrenia: challenges and management strategies. Patient Relat Outcome Meas.

[22] Steinkamp JM, Goldblatt N, Borodovsky JT, LaVertu A, Kronish IM, Marsch LA, Schuman-Olivier Z (2019). Technological interventions for medication adherence in adult mental health and substance use disorders: a systematic review. JMIR Ment Health.

[23] Cullen BA, McGinty EE, Zhang Y, Dosreis SC, Steinwachs DM, Guallar E, Daumit GL (2013). Guideline-concordant antipsychotic use and mortality in schizophrenia. Schizophr Bull.

[24] Higashi K, Medic G, Littlewood KJ, Diez T, Granström Ola, De Hert M (2013). Medication adherence in schizophrenia: factors influencing adherence and consequences of nonadherence, a systematic literature review. Ther Adv Psychopharmacol.

[25] Kane JM, Perlis RH, DiCarlo LA, Au-Yeung K, Duong J, Petrides G (2013). First experience with a wireless system incorporating physiologic assessments and direct confirmation of digital tablet ingestions in ambulatory patients with schizophrenia or bipolar disorder. J Clin Psychiatry.

[26] Knights J, Heidary Z, Peters-Strickland T, Ramanathan M (2019). Evaluating digital medicine ingestion data from seriously mentally ill patients with a Bayesian Hybrid Model. NPJ Digit Med.

[27] Fowler JC, Cope N, Knights J, Phiri P, Makin A, Peters-Strickland T, Rathod S (2019). Hummingbird study: a study protocol for a multicentre exploratory trial to assess the acceptance and performance of a digital medicine system in adults with schizophrenia, schizoaffective disorder or first-episode psychosis. BMJ Open.

[28] Cohen EA, Skubiak T, Hadzi Boskovic D, Norman K, Knights J, Fang H, Coppin-Renz A, Peters-Strickland T, Lindenmayer JP, Reuteman-Fowler JC (2022). Phase 3b multicenter, prospective, open-label trial to evaluate the effects of a digital medicine system on inpatient psychiatric hospitalization rates for adults with schizophrenia. J Clin Psychiatry.

[29] Fowler JC, Cope N, Knights J, Fang H, Skubiak T, Shergill SS, Phiri P, Rathod S, Peters-Strickland T (2021). Hummingbird study: results from an exploratory trial assessing the performance and acceptance of a digital medicine system in adults with schizophrenia, schizoaffective disorder, or first-episode psychosis. Neuropsychiatr Dis Treat.

[30] Rohatagi S, Profit D, Hatch A, Zhao C, Docherty JP, Peters-Strickland TS (2016). Optimization of a digital medicine system in psychiatry. J Clin Psychiatry.

[31] Peters-Strickland T, Pestreich L, Hatch A, Rohatagi S, Baker RA, Docherty JP, Markovtsova L, Raja P, Weiden PJ, Walling DP (2016). Usability of a novel digital medicine system in adults with schizophrenia treated with sensor-embedded tablets of aripiprazole. Neuropsychiatr Dis Treat.

[32] Kopelowicz A, Baker RA, Zhao C, Brewer C, Lawson E, Peters-Strickland T (2017). A multicenter, open-label, pilot study evaluating the functionality of an integrated call center for a digital medicine system to optimize monitoring of adherence to oral aripiprazole in adult patients with serious mental illness. Neuropsychiatr Dis Treat.

[33] Profit D, Rohatagi S, Zhao C, Hatch A, Docherty JP, Peters-Strickland TS (2016). Developing a digital medicine system in psychiatry: ingestion detection rate and latency period. J Clin Psychiatry.

[34] (2019). A phase IIIb multi-center, open-label, mirror-image, trial in adult subjects with schizophrenia treated prospectively for 6-months with Abilify MyCite®. Otsuka.

[35] Clinical trial data transparency. Otsuka.

