# Supplementary material for: Safety Evaluation in Iterative Development of Wearable Patches for Aripiprazole Tablets With Sensor: Pooled Analysis of Clinical Trials
Source: JMIR Form Res. 2023 Dec 12;7:e44768. doi: 10.2196/44768 (PMC10751624; doi:10.2196/44768)
Supplement: Multimedia Appendix 1 [file formative_v7i1e44768_app1.docx]

| Trial identifiers | | Duration | Participants, n | Wearable patch(es) used |
| --- | --- | --- | --- | --- |
| **Long-term studies**^a^ | |  |  |  |
|  | 316-13-204 | 16 weeks | 58 | RP4 |
|  | 316-13-205 | 4 weeks | 30 | RP4 |
|  | 316-13-215^b^ | 8 weeks | 49 | DW5 |
|  | 316-14-220^c^ | 8 weeks | 67 | RP4, DW5 |
|  | 031-201-00186^d^ | 8 weeks | 43 | DW5 |
|  | 031-201-00301^e^ | 6 months | 277 | DW5, RW2 |
| **Short-term studies**^f^ | |  |  |  |
|  | 316-13-206A^g^ | <1 day | 30 | RP4 |
|  | 316-13-206B^g^ | <1 day | 29 | DW5 |
|  | 031-201-00266 | 8 days | 80 | RW2 |
|  | 031-201-00383 | 10 days | 39 | DW5, RW2 |
|  | 031-201-00420 | 10 days | 46 | DW5, RW2 |
|  | 031-201-00469 | 10 days | 15 | RW2^h^ |

^a^In long-term studies, participants were exposed to a wearable patch multiple times successively as participants wore one wearable patch at a time and were instructed to replace the wearable patch every 7 days.

^b^ClinicalTrials.gov identifier: NCT02722967.

^c^ClinicalTrials.gov identifier: NCT02219009.

^d^ClinicalTrials.gov identifier: NCT03568500.

^e^ClinicalTrials.gov identifier: NCT03892889.

^f^In short-term studies, participants wore one or multiple wearable patches simultaneously on distinct parts of abdomen for the whole study duration without replacement of wearable patches.

^g^ClinicalTrials.gov identifier: NCT02091882.

^h^Participants also received prototype wearable patches of AS simultaneously.

DW5, disposable wearable sensor version 5; RW2, reusable wearable sensor version 2; RP4, raisin patch version 4.
