# Supplementary material for: Safety Evaluation in Iterative Development of Wearable Patches for Aripiprazole Tablets With Sensor: Pooled Analysis of Clinical Trials
Source: JMIR Form Res. 2023 Dec 12;7:e44768. doi: 10.2196/44768 (PMC10751624; doi:10.2196/44768)
Supplement: Multimedia Appendix 2 [file formative_v7i1e44768_app2.docx]

|  | | RP4 | DW5 | RW2 | Any wearable patch |
| --- | --- | --- | --- | --- | --- |
| **316-13-204**^a^ | | | | | |
| Number of participants | | 58 | – | – | 58 |
| Any SIEs, n (%) | | 15 (25.9) | – | – | 15 (25.9) |
| **Application site** | |  |  |  |  |
|  | Erythema | 4 (6.9) | – | – | 4 (6.9) |
|  | Pain | 1 (1.7) | – | – | 1 (1.7) |
|  | Pruritus | 6 (10.3) | – | – | 6 (10.3) |
|  | Rash | 4 (6.9) | – | – | 4 (6.9) |
| Rash | | 1 (1.7) | – | – | 1 (1.7) |
| Pruritus | | 3 (5.2) | – | – | 3 (5.2) |
| Contact dermatitis | | 1 (1.7) | – | – | 1 (1.7) |
| **316-13-205**^a^ | | | | | |
| Number of participants | | 30 | – | – | 30 |
| Any SIEs, n (%) | | 0 (0.0) | – | – | 0 (0.0) |
| **316-13-206A**^a,b^ | | | | | |
| Number of participants | | 30 | – | – | 30 |
| Any SIEs, n (%) | | 0 (0.0) | – | – | 0 (0.0) |
| **316-13-206B**^a,b^ | | | | | |
| Number of participants | | – | 29 | – | 29 |
| Any SIEs, n (%) | | – | 0 (0.0) | – | 0 (0.0) |
| **316-13-215**^a,c^ | | | | | |
| Number of participants | | – | 49 | – | 49 |
| Any SIEs, n (%) | | – | 16 (32.7) | – | 16 (32.7) |
| Rash | | – | 11 (22.4) | – | 11 (22.4) |
| Pruritus | | – | 2 (4.1) | – | 2 (4.1) |
| Erythema | | – | 2 (4.1) | – | 2 (4.1) |
| Skin irritation | | – | 1 (2.0) | – | 1 (2.0) |
| **316-14-220**^a,d^ | | | | | |
| Number of participants | | 37 | 30 | – | 67 |
| Any SIEs, n (%) | | 13 (35.1) | 9 (30.0) | – | 22 (32.8) |
| **Rash**^e^ | |  |  |  |  |
|  | Erythematous | 2 (5.4) | 0 (0.0) | – | 2 (3.0) |
|  | Papular | 1 (2.7) | 1 (3.3) | – | 2 (3.0) |
|  | Pruritic | 0 (0.0) | 1 (3.3) | – | 1 (1.5) |
|  | Not specified | 2 (5.4) | 0 (0.0) | – | 2 (3.0) |
| Pruritus | | 5 (13.5) | 4 (13.3) | – | 9 (13.4) |
| Blister | | 1 (2.7) | 0 (0.0) | – | 1 (1.5) |
| Contact dermatitis | | 2 (5.4) | 1 (3.3) | – | 3 (4.5) |
| Erythema | | 2 (5.4) | 2 (6.7) | – | 4 (6.0) |
| Skin abrasion | | 1 (2.7) | 0 (0.0) | – | 1 (1.5) |
| Skin discoloration | | 0 (0.0) | 1 (3.3) | – | 1 (1.5) |
| Skin hyperpigmentation | | 0 (0.0) | 1 (3.3) | – | 1 (1.5) |
| Skin irritation | | 0 (0.0) | 1 (3.3) | – | 1 (1.5) |
| **031-201-00186**^a,f^ | | | | | |
| Number of participants | | – | 43 | – | 43 |
| Any SIEs, n (%) | | – | 9 (20.9) | – | 9 (20.9) |
| **Medical device site** | |  |  |  |  |
|  | Irritation | – | 9 (20.9) | – | 9 (20.9) |
| **031-201-00266**^a^ | | | | | |
| Number of participants | | – | – | 80 | 80 |
| Any SIEs, n (%) | | – | – | 7 (8.8) | 7 (8.8) |
| **Medical device site** | |  |  |  |  |
|  | Erythema | – | – | 3 (3.8) | 3 (3.8) |
|  | Irritation | – | – | 2 (2.5) | 2 (2.5) |
|  | Pruritus | – | – | 1 (1.3) | 1 (1.3) |
|  | Rash | – | – | 1 (1.3) | 1 (1.3) |
| **031-201-00383**^a,g^ | | | | | |
| Number of participants | | – | 39 | 39 | 39 |
| Any SIEs, n (%) | | – | 0 (0.0) | 2 (5.1) | 2 (5.1) |
| **Medical device site** | |  |  |  |  |
|  | Erythema | – | 0 (0.0) | 1 (2.6) | 1 (2.6) |
|  | Pruritus | – | 0 (0.0) | 1 (2.6) | 1 (2.6) |
|  | Rash | – | 0 (0.0) | 1 (2.6) | 1 (2.6) |
| **031-201-00420**^a,g^ | | | | | |
| Number of participants | | – | 46 | 46 | 46 |
| Any SIEs, n (%) | | – | 7 (15.2) | 10 (21.7) | 10 (21.7) |
| **Medical device site** | |  |  |  |  |
|  | Erythema | – | 1 (2.2) | 1 (2.2) | 1 (2.2) |
|  | Pruritus | – | 6 (13.0) | 9 (19.6) | 9 (19.6) |
|  | Reaction | – | 1 (2.2) | 1 (2.2) | 1 (2.2) |
| **031-201-00301**^a,h^ | | | | | |
| Number of participants | | – | 151 | 126 | 277 |
| Any SIEs, n (%) | | – | 14 (9.3) | 7 (5.6) | 21 (7.6) |
| **Medical device site** | |  |  |  |  |
|  | Erythema | – | 0 (0.0) | 1 (0.8) | 1 (0.4) |
|  | Irritation | – | 7 (4.6) | 0 (0.0) | 7 (2.5) |
|  | Pruritus | – | 2 (1.3) | 4 (3.2) | 6 (2.2) |
|  | Rash | – | 6 (4.0) | 2 (1.6) | 8 (2.9) |
|  | Reaction | – | 0 (0.0) | 1 (0.8) | 1 (0.4) |
| **031-201-00469**^a^ | | | | | |
| Number of participants | | – | – | 15 | 15 |
| Any SIEs, n (%) | | – | – | 2 (13.3) | 2 (13.3) |
| **Medical device site** | |  |  |  |  |
|  | Irritation | – | – | 2 (13.3) | 2 (13.3) |

^a^Trial identifiers.

^b^ClinicalTrials.gov identifier: NCT02091882.

^c^ClinicalTrials.gov identifier: NCT02722967.

^d^ClinicalTrials.gov identifier: NCT02219009.

^e^Adverse events reported from earlier clinical trials with RP4 and DW5 were coded to specify types of rash and were evolved to specify medical device site in later trials with DW5 and RW2.

^f^ClinicalTrials.gov identifier: NCT03568500.

^g^Eighty-five participants were exposed to multiple wearable patches and experienced events with different wearable patches, thus, the sum of the DW5 and RW2 counts may not be equal to the any wearable patch counts.

^h^ClinicalTrials.gov identifier: NCT03892889.

DW5, disposable wearable sensor version 5; RW2, reusable wearable sensor version 2; RP4, raisin patch version 4; SIEs, skin irritation events.
