# Supplementary material for: Safety Evaluation in Iterative Development of Wearable Patches for Aripiprazole Tablets With Sensor: Pooled Analysis of Clinical Trials
Source: JMIR Form Res. 2023 Dec 12;7:e44768. doi: 10.2196/44768 (PMC10751624; doi:10.2196/44768)
Supplement: Multimedia Appendix 3 [file formative_v7i1e44768_app3.docx]

|  | | RP4 | DW5 | RW2 | Any wearable patch |
| --- | --- | --- | --- | --- | --- |
| **316-13-215**^a,b^ | | | | | |
| Number of participants | | – | 49 | – | 49 |
| Wearable patch use years (days) | | – | 5.7 (2093) | – | 5.7 (2093) |
| Any SIEs, n (incidence rate^c^) | | – | 16 (2.792) | – | 16 (2.792) |
| Erythema | | – | 2 (0.349) | – | 2 (0.349) |
| Rash | | – | 11 (1.920) | – | 11 (1.920) |
| Pruritus | | – | 2 (0.349) | – | 2 (0.349) |
| Skin irritation | | – | 1 (0.175) | – | 1 (0.175) |
| **316-14-220**^a,d^ | | | | | |
| Number of participants | | 37 | 30 | – | 67 |
| Wearable patch use years (days) | | 4.1 (1495) | 2.9 (1041) | – | 6.9 (2536) |
| Any SIEs, n (incidence rate^c^) | | 13 (3.176) | 9 (3.158) | – | 22 (3.169) |
| Blister | | 1 (0.244) | 0 (0.000) | – | 1 (0.144) |
| Contact dermatitis | | 2 (0.489) | 1 (0.351) | – | 3 (0.432) |
| Erythema | | 2 (0.489) | 2 (0.702) | – | 4 (0.576) |
| **Rash**^e^ | |  |  |  |  |
|  | Erythematous | 2 (0.489) | 0 (0.000) | – | 2 (0.288) |
|  | Papular | 1 (0.244) | 1 (0.351) | – | 2 (0.288) |
|  | Pruritic | 0 (0.000) | 1 (0.351) | – | 1 (0.144) |
|  | Not specified | 2 (0.489) | 0 (0.000) | – | 2 (0.288) |
| Pruritus | | 5 (1. 222) | 4 (1.403) | – | 9 (1.296) |
| Skin abrasion | | 1 (0.244) | 0 (0.000) | – | 1 (0.144) |
| Skin discoloration | | 0 (0.000) | 1 (0.351) | – | 1 (0.144) |
| Skin hyperpigmentation | | 0 (0.000) | 1 (0.351) | – | 1 (0.144) |
| Skin irritation | | 0 (0.000) | 1 (0.351) | – | 1 (0.144) |
| **031-201-00186**^a,f^ | | | | | |
| Number of participants | | – | 43 | – | 43 |
| Wearable patch use years (days) | | – | 3.3 (1195) | – | 3.3 (1195) |
| Any SIEs, n (incidence rate^c^) | | – | 9 (2.751) | – | 9 (2.751) |
| **Medical device site** | |  |  |  |  |
|  | Irritation | – | 9 (2.751) | – | 9 (2.751) |
| **031-201-00301**^a,g^ | | | | | |
| Number of participants | | – | 151 | 126 | 277 |
| Wearable patch use years (days) | | – | 18.3 (6679) | 5.3 (1938) | 23.6 (8617) |
| Any SIEs, n (incidence rate^c^) | | – | 14 (0.766) | 7 (1.319) | 21 (0.890) |
| **Medical device site** | |  |  |  |  |
|  | Erythema | – | 0 (0.000) | 1 (0.188) | 1 (0.042) |
|  | Irritation | – | 7 (0.383) | 0 (0.000) | 7 (0.297) |
|  | Pruritus | – | 2 (0.109) | 4 (0.754) | 6 (0.254) |
|  | Rash | – | 6 (0.328) | 2 (0.377) | 8 (0.339) |
|  | Reaction | – | 0 (0.000) | 1 (0.188) | 1 (0.042) |

^a^Trial identifiers.

^b^ClinicalTrials.gov identifier: NCT02722967.

^c^Per person-year.

^d^ClinicalTrials.gov identifier: NCT02219009.

^e^Adverse events reported from earlier clinical trials with RP4 and DW5 were coded to specify types of rash and were evolved to specify medical device site in later trials with DW5 and RW2.

^f^ClinicalTrials.gov identifier: NCT03568500.

^g^ClinicalTrials.gov identifier: NCT03892889.

DW5, disposable wearable sensor version 5; RW2, reusable wearable sensor version 2; RP4, raisin patch version 4; SIEs, skin irritation events.
