# Supplementary material for: Safety Evaluation in Iterative Development of Wearable Patches for Aripiprazole Tablets With Sensor: Pooled Analysis of Clinical Trials
Source: JMIR Form Res. 2023 Dec 12;7:e44768. doi: 10.2196/44768 (PMC10751624; doi:10.2196/44768)
Supplement: Multimedia Appendix 4 [file formative_v7i1e44768_app4.docx]

|  | | RP4 | DW5 | RW2 | Any wearable patch |
| --- | --- | --- | --- | --- | --- |
| **031-201-00266**^a^ | | | | | |
| Number of participants | | – | – | 80 | 80 |
| Wearable patch use years (days) | | – | – | 1.8 (669) | 1.8 (669) |
| Any SIEs, n (incidence rate^b^) | | – | – | 7 (3.822) | 7 (3.822) |
| **Medical device site** | |  |  |  |  |
|  | Erythema | – | – | 3 (1.638) | 3 (1.638) |
|  | Irritation | – | – | 2 (1.092) | 2 (1.092) |
|  | Pruritus | – | – | 1 (0.546) | 1 (0.546) |
|  | Rash | – | – | 1 (0.546) | 1 (0.546) |
| **031-201-00383**^a,c^ | | | | | |
| Number of participants | | – | 39 | 39 | 39 |
| Wearable patch use years (days) | | – | 1.1 (390) | 1.1 (390) | 1.1 (390) |
| Any SIEs, n (incidence rate^b^) | | – | 0 (0.000) | 2 (1.873) | 2 (1.873) |
| **Medical device site** | |  |  |  |  |
|  | Erythema | – | 0 (0.000) | 1 (0.937) | 1 (0.937) |
|  | Pruritus | – | 0 (0.000) | 1 (0.937) | 1 (0.937) |
|  | Rash | – | 0 (0.000) | 1 (0.937) | 1 (0.937) |
| **031-201-00420**^a,c^ | | | | | |
| Number of participants | | – | 46 | 46 | 46 |
| Wearable patch use years (days) | | – | 1.3 (460) | 1.3 (460) | 1.3 (460) |
| Any SIEs, n (incidence rate^b^) | | – | 7 (5.558) | 10 (7.940) | 10 (7.940) |
| **Medical device site** | |  |  |  |  |
|  | Erythema | – | 1 (0.794) | 1 (0.794) | 1 (0.794) |
|  | Pruritus | – | 6 (4.764) | 9 (7.146) | 9 (7.146) |
|  | Reaction | – | 1 (0.794) | 1 (0.794) | 1 (0.794) |
| **031-201-00469**^a^ | | | | | |
| Number of participants | | – | – | 15 | 15 |
| Wearable patch use years (days) | | – | – | 0.4 (150) | 0.4 (150) |
| Any SIEs, n (incidence rate^b^) | | – | – | 2 (4.870) | 2 (4.870) |
| **Medical device site** | |  |  |  |  |
|  | Irritation | – | – | 2 (4.870) | 2 (4.870) |

^a^Trial identifiers.

^b^Per person-year.

^c^Eighty-five participants were exposed to multiple wearable patches and experienced events with different wearable patches, thus, the sum of the DW5 and RW2 counts may not be equal to the any wearable patch counts.

DW5, disposable wearable sensor version 5; RW2, reusable wearable sensor version 2; RP4, raisin patch version 4; SIEs, skin irritation events.
