# Supplementary material for: Safety Evaluation in Iterative Development of Wearable Patches for Aripiprazole Tablets With Sensor: Pooled Analysis of Clinical Trials
Source: JMIR Form Res. 2023 Dec 12;7:e44768. doi: 10.2196/44768 (PMC10751624; doi:10.2196/44768)
Supplement: Multimedia Appendix 5 [file formative_v7i1e44768_app5.docx]

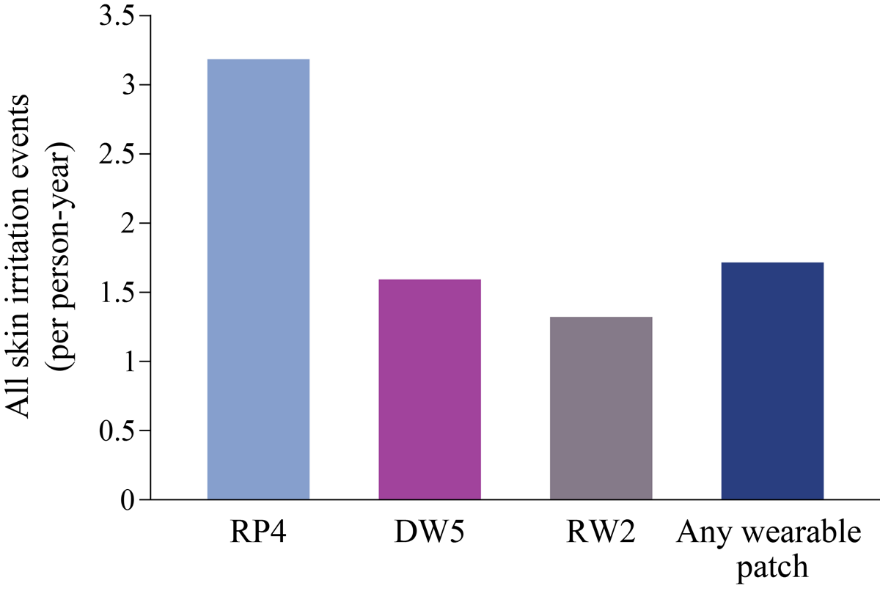


Long-term studies (≥ 4 weeks duration) with available data were included (Trial identifiers [trial registration numbers]: 316-13-215 [NCT02722967], 316-14-220 [NCT02219009], 031-201-00186 [NCT03568500], 031-201-00301 [NCT03892889]).

DW5, disposable wearable sensor version 5. RW2, reusable wearable sensor version 2. RP4, raisin patch version 4. SIEs, skin irritation events
